# Recent Developments in Sonochemical Synthesis of Nanoporous Materials

**DOI:** 10.3390/molecules28062639

**Published:** 2023-03-14

**Authors:** Sylwia Głowniak, Barbara Szczęśniak, Jerzy Choma, Mietek Jaroniec

**Affiliations:** 1 Institute of Chemistry, Military University of Technology, Kaliskiego 2, 00-908 Warsaw, Poland; sylwia.glowniak@wat.edu.pl (S.G.); barbara.szczesniak@wat.edu.pl (B.S.); jerzy.choma@wat.edu.pl (J.C.); 2 Department of Chemistry and Biochemistry, Kent State University, Kent, OH 44242, USA

**Keywords:** ultrasounds, sonochemical synthesis, porous materials, cavitation, mechanochemical synthesis

## Abstract

Ultrasounds are commonly used in medical imaging, solution homogenization, navigation, and ranging, but they are also a great energy source for chemical reactions. Sonochemistry uses ultrasounds and thus realizes one of the basic concepts of green chemistry, i.e., energy savings. Moreover, reduced reaction time, mostly using water as a solvent, and better product yields are among the many factors that make ultrasound-induced reactions greener than those performed under conventional conditions. Sonochemistry has been successfully implemented for the preparation of various materials; this review covers sonochemically synthesized nanoporous materials. For instance, sonochemical-assisted methods afforded ordered mesoporous silicas, spherical mesoporous silicas, periodic mesoporous organosilicas, various metal oxides, biomass-derived activated carbons, carbon nanotubes, diverse metal-organic frameworks, and covalent organic frameworks. Among these materials, highly porous samples have also been prepared, such as garlic peel-derived activated carbon with an apparent specific surface area of 3887 m^2^/g and MOF-177 with an SSA of 4898 m^2^/g. Additionally, many of them have been examined for practical usage in gas adsorption, water treatment, catalysis, and energy storage-related applications, yielding satisfactory results.

## 1. Introduction

### 1.1. What Is Sonochemical Synthesis?

**Figure 1:**
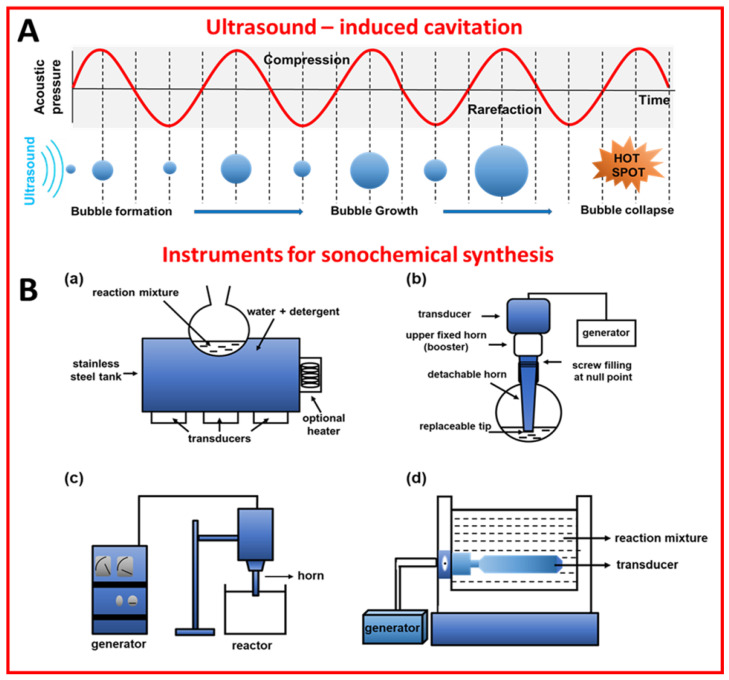
(**A**) Schematic illustration of ultrasound-induced cavitation [6]. Reproduced with permission from ref. [6], licensed under CC-BY. (**B**) Schematic illustration of the instruments for sonochemical synthesis: (**a**) cleaning bath [12], (**b**) probe system [12], (**c**) horn reactor [14], and (**d**) longitudinal horn reactor [14]. Reproduced with permission from ref. [12]. Copyright 2002, Blackwell Science Ltd. Reproduced with permission from ref. [14]. Copyright 2011, Elsevier B.V.

Sonochemical synthesis relies on ultrasound-induced cavitation, namely the generation, growth, and collapse of bubbles to induce chemical reactions [1,2]. Ultrasounds (US) are a form of electromagnetic energy with frequencies ranging from 20 kHz to 1 MHz [2]. When the reaction mixture is treated with ultrasounds, which are actually pressure waves, the gases and particles present in the solution are compressed and expanded alternately. During expansion, the intermolecular forces of the liquid are overloaded, and cavitation bubbles can be formed. The bubbles grow and accumulate energy until they reach a critical size, then the bubbles collapse and the stored energy is released in a very short time (Figure 1A). This phenomenon (called cavitation) results in an extreme local temperature and pressure (“hot spots”) of even ~5300 °C and ∼1000 atmospheres, respectively [3]. It should be emphasized that there is no direct interaction between ultrasounds and chemical species at a molecular level, and the energy required for the synthesis is delivered by ultrasound-induced cavitation [4]. One of the effects of cavitation is the formation of radicals that are responsible for the initiation of chemical reactions. Physical/mechanical effects accompanying cavitation, namely heating, microjet, and shockwave, play a crucial role in chemical syntheses, e.g., accelerate mass transport, have an impact on morphologies and surface composition, as well as generate nanostructures, e.g., exfoliate layered materials into 2D layered ones [3]. For a detailed explanation, readers are referred to refs [3,5,6].

### 1.2. History of Sonochemistry

The beginning of using ultrasound dates to 1883, when Francis Galton invented the silent whistle, emitting sounds heard only by dogs that were exactly ultrasounds [7]. Then, in 1895, Thornycroft and Barnaby delivered the first report on cavitation after they had observed erosion and damage to the propeller of their submarine. In 1917, Lord Rayleigh determined the first mathematical model describing cavitation in an incompressible fluid [7,8]. Meanwhile, the tragedy of the Titanic in 1912 prompted Paul Langevin to develop a hydrophone, i.e., a device that uses ultrasounds to detect submarines [7]. A breakthrough in ultrasound-devoted research was made in 1927 by three scientists: Alfred Lee Loomis, Robert William Wood, and Theodore William Richards, who discovered the chemical and biological effects caused by ultrasounds [7,9]. The following years were abundant in different developments in the field of ultrasounds. The most important achievements are the first computer modeling of a cavitating bubble (Noltingk and Neppiras, 1950), the sonolysis of an organic liquid (Schultz and Henglein, 1953), the attempt to explain ultrasonic cleaning effects in heterogeneous systems (Elder et al., 1954), the first marketed ultrasonic generators (1980), and the introduction of the term “sonochemistry” in the literature for the first time (Ernest Arthur Neppiras, 1980), among others [7,9,10]. While ultrasounds have been used for decades, the history of sonochemistry is relatively recent. At the turn of the 1980s and 1990s, the first commercial devices for sonochemical research were created. In 1987, the Sonochemistry Group was founded to develop methods to facilitate the use of ultrasounds in industry. A few years later (in 1994), the journal dedicated to the applications of ultrasounds in chemistry—“Ultrasonics Sonochemistry”—was established [9].

### 1.3. Sonochemical Equipment

There are a few types of reactors for ultrasound-assisted syntheses, such as ultrasonic baths, ultrasonic probes (horns), longitudinal horns, and multiple transducers [11]. These reactors differ in the way they introduce ultrasounds into a reacting system. For instance, ultrasonic baths introduce energy into the system through water and reaction vessel walls, while ultrasonic probes introduce the energy directly into the system, which is more desirable when localized energy is required [12]. Multiple transducers, as well as longitudinal horns, are preferable for large-scale applications [11]. Schemes of the sonochemical reactors are presented in Figure 1B. The basic element of sonochemical devices is a transducer that converts mechanical or electrical energy into ultrasounds [12]. The most common are piezoelectric transducers made of barium titanate, lead methaniobate, or other piezoelectric materials. They can work in the entire range of ultrasonic frequencies, which is the key parameter influencing the course of the synthesis. Namely, high frequencies (above 100 kHz) are appropriate for the chemical effect, while the lower frequencies are preferable for physical effects [11]. It should be remembered that there are many other important parameters affecting the sonochemical synthesis, including mass transfer, mixing time, flow diagram, and solvent type [13,14]. For example, solvents with low surface tension support bubble growth, though they reduce cavitation intensity [8].

### 1.4. Comparison of Ultrasound-Assisted and Other Mechanochemical Syntheses

A mechanochemical reaction is defined by IUPAC as “a chemical reaction that is induced by the direct absorption of mechanical energy” [15]. The delivery of the mechanical energy usually relies on direct mechanical effects on reactants through grinding, ball- or pan-milling, shearing, compression, etc. [16]. Such actions induce plastic and elastic deformations, defects, and other surface changes, resulting in chemical bond breakage and new bond formation. Ultrasonically induced cavitation leads to similar physical and chemical consequences, i.e., local high pressure and temperature, crystal deformation, shear stresses, acceleration of diffusion processes, breaking of chemical bonds, formation of highly reactive radicals, etc. [17,18]. It is because ultrasounds produce some physical effects (e.g., shock waves), which then mechanically affect the reaction system. For example, ultrasound-induced waves accelerate solid particles suspended in the liquid and evoke changes in their morphologies, crystallinity, and surface compositions, among others [17].

Undoubtedly, non-conventional energy sources such as ultrasounds, microwaves, and ball milling have many benefits in terms of green chemistry and environmentally friendly synthesis procedures, especially energy- and time-savings. Nevertheless, there are some differences between these methods that contribute to their effectiveness. The advantage of ball milling is a significant reduction (or elimination) of solvent consumption and a shortening of reaction times and synthesis steps. However, it should be remembered that the use of this approach may result in the amorphization of crystalline products, contamination of products related to the mechanical contact of the balls with the ground substrates, as well as other side effects associated with the incomplete control over the whole milling process. Microwave-assisted synthesis provides fast, uniform, and/or selective heating, maintaining the high purity of products, but it requires a careful selection of reagents, i.e., those that have specific dielectric properties. Moreover, microwave photons supply energy to the reaction system that is insufficient to break chemical bonds. In turn, ultrasound-assisted syntheses benefit from the combined high energy input and intensive mechanical effects provided by the above-described ultrasound-induced cavitation. Apparently, using ultrasounds affords higher temperatures in a shorter time while maintaining high heat uniformity in comparison to other heating methods. It is our belief that these features may become a gateway to the synthesis of new advanced materials having superior properties.

### 1.5. Main Groups of Sonochemically Synthesized Nanoporous Materials

**Figure 2:**
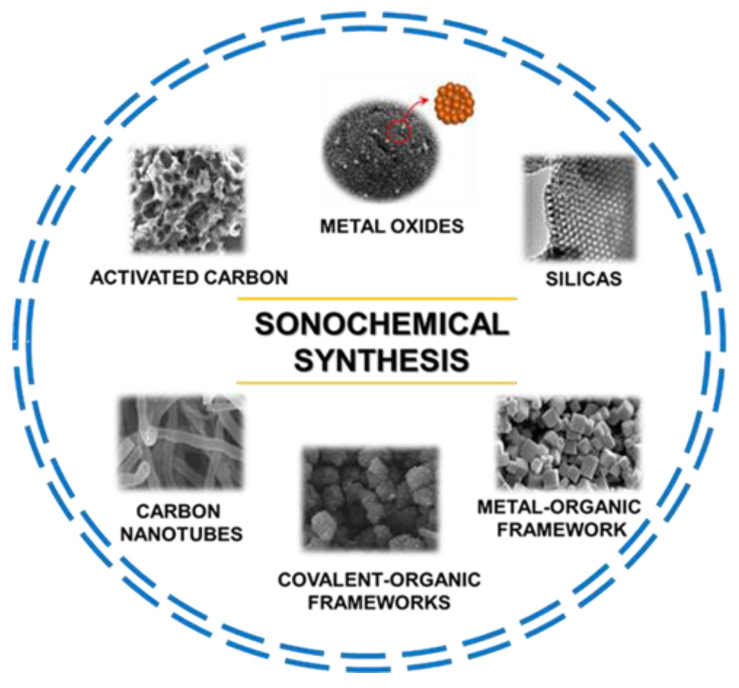
Schematic illustration of diverse nanoporous materials synthesized via a sonochemical method including their scanning electron microscopy (SEM) images. Image of metal oxides reproduced with permission from Sankar et al. Ceram. Int. 2018, 44, 17514–17521 (Copyright 2018, Elsevier Ltd. and Techna Group S.r.I.); image of silicas reproduced with permission from Palani et al. Microporous Mesoporous Mater. 2010, 131, 385–392 (Copyright 2010, Elsevier Inc.); image of metal-organic frameworks reproduced with permission from Yu et al. Microporous Mesoporous Mater. 2021, 316, 110985 (Copyright 2021, Elsevier Inc.); image of covalent-organic frameworks reproduced with permission from Duan et al. J. Membrane. Sci. 2019, 572, 588–595 (Copyright 2018, Elsevier B.V.); image of carbon nanotubes reproduced with permission from Liu et al. Carbon 2017, 115, 754–762 (Copyright 2017, Elsevier Ltd.); image of activated carbon reproduced with permission from Ghani et al. J. Colloid Interface Sci. 2022, 611, 578–587 (Copyright 2021, Elsevier Inc.).

There are enormous opportunities in using ultrasounds in the synthesis of many different functional nanoporous materials with potential applications in many areas. This is not surprising, considering the current green chemistry trend that emphasizes replacing conventional energy sources with more environmentally friendly ones, such as ultrasounds and microwaves, among others. The range of nanoporous materials obtained via the sonochemical route includes silicas, organosilicas, metal oxides, carbons, metal–organic frameworks (MOFs), covalent-organic frameworks (COFs), and countless composites of various materials. Figure 2 shows the main groups of nanoporous materials synthesized via sonochemically assisted methods.

## 2. Sonochemical Synthesis, Properties, and Applications of Nanoporous Materials

Nanoporous materials refer to organic and inorganic structures with pore sizes between 1 and 100 nm. According to the classification provided by IUPAC, nanoporous materials are divided into microporous (pore size below 2 nm), mesoporous (pore size between 2 and 50 nm), and macroporous (pore size between 50 and 100 nm) materials [19]. The most important parameters of porosity are: (i) specific surface area (SSA, often calculated by using the Brunauer–Emmett–Teller model, BET); (ii) total pore volume (V_t_); and (iii) pore size distribution (PSD), which are usually determined based on low-temperature nitrogen adsorption isotherms. Most often, practical applications of porous materials require their high porosity (large SSA values). However, high SSA is a relative term; for example, highly porous activated carbons possess the apparent SSA even above 3000 m^2^/g, while high SSA for metal oxides is below 1000 m^2^/g. High porosity is particularly important in gas adsorption and storage, wastewater treatment, electrochemical applications (supercapacitors, batteries, oxygen reduction reactions, etc.), catalysis, and drug delivery. Apart from porosity, other physicochemical properties and characteristics (e.g., morphology, thermal stability, crystallinity) have a great impact on the potential applications of the materials; thus, we also include such features in the review.

### 2.1. Silicas and Organosilicas

**Figure 3:**
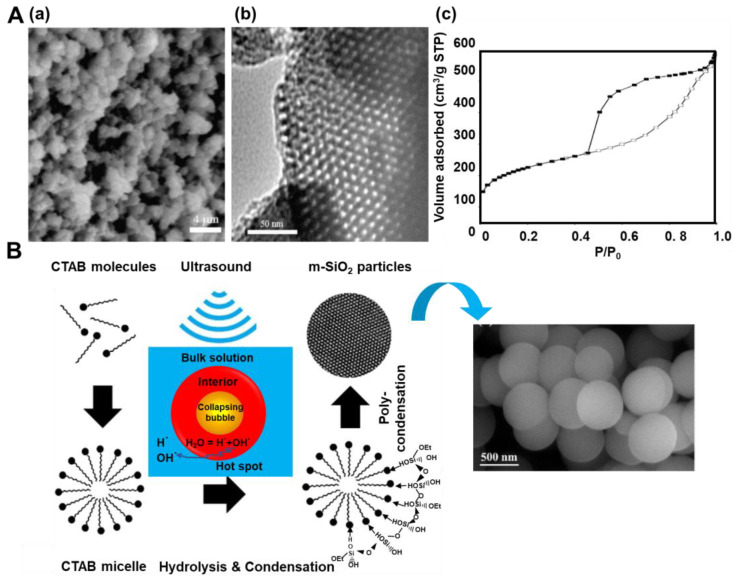
(**A**) (**a**) SEM images, (**b**) TEM images and (**c**) nitrogen adsorption-desorption isotherm of SBA-15 sonicated at 80 °C [22]. Reproduced with permission from ref. [22]. Copyright 2010, Elsevier Inc. (**B**) Mechanism of formation of spherical mesoporous silica under ultrasound and SEM images of the spheres [23]. Reproduced with permission from ref. [23]. Copyright 2020, Elsevier B.V.

The synthesis of silicas most often involves a sol-gel method relying on the hydrolysis of a silica precursor (e.g., tetraethylorthosilicate—TEOS), which then undergoes condensation, leading to polymeric networks. Incorporating some additives (usually organic molecules) during the synthesis may result in some changes in the product structure or even an entirely different product. For example, ordered mesoporous silicas (OMSs) are formed by the addition of a surfactant or polymeric template, e.g., CTAB (cetyltrimethylammonium bromide) or Pluronic P123 (EO_20_PO_70_EO_20_). As mentioned above, sonochemistry has been successfully implemented in the synthesis of OMS. For instance, Vetrivel et al. [20] synthesized MCM-41 (MCM—Mobil Composition of Matter)—an ordered mesoporous material with a hexagonal arrangement of cylindrical pores. The procedure relied on the sonication of the solution of CTAB, NH_4_OH, TEOS, and water, followed by calcination at 550 °C for 4 h in air. The TEOS:CTAB:NH_4_OH:H_2_O molar ratio was 1:0.125:69:525. A high surface area sample with SSA of 1662 m^2^/g and V_t_ of 0.89 cm^3^/g was obtained after sonication for only 5 min. X-ray diffraction (XRD) patterns and transmission electron microscopy (TEM) images confirmed its highly ordered hexagonal mesoporous structure. Ultrasound waves were also used to remove surfactant templates from mesostructured MCM-41 silicas [21]. This approach resulted in silicas with higher values of structural parameters than those obtained using a conventional calcination process. For instance, MCM-41 calcined in air at 550 °C for 5 h possessed an SSA of 1276 m^2^/g and a V_t_ of 0.45 cm^3^/g, while MCM-41 sonicated in ethanol at 60 °C for 30 min and possessed an SSA of 1325 m^2^/g and a V_t_ of 0.47 cm^3^/g. The authors reported that the majority (93%) of CTAB template molecules could be removed from the dried template-containing MCM-41 silica within only 15 min during sonication (at 28 kHz and 40 °C) in an alcoholic solution. This study proved that ultrasonic treatments could be successfully utilized to separate and purify silica products, which seems to be an easy, cost-effective, and mild method for template removal compared to the calcination method. Silica SBA-15, similar to MCM-41, has a hexagonal mesostructure but with thicker walls and a different pore diameter (Figure 3A). Palani et al. [22] obtained SBA-15 silicas with SSA up to 717 m^2^/g, V_t_ up to 0.84 cm^3^/g, and pore diameters in the range of 4.1–4.9 nm. The synthesis gel composition was comprised of TEOS, Pluronic P123, HCl, and water in a molar ratio of 1:0.0167:5.82:190, respectively. Such a mixture was held under ultrasound for 1 h at a temperature ranging from 40 to 100 °C prior to stirring at room temperature for 2 h, and then it was calcined at 550 °C for 6 h. The highest SSA value (717 m^2^/g) was achieved for silica obtained by applying sonication at 40 °C for 1 h. Varying the temperature and time of ultrasound treatments resulted in some changes to the structural properties of the final SBA-15 products. Increasing the reaction temperature from 40 to 80 °C is manifested in an enhanced hydrolysis rate of TEOS and weaker interactions with the P123 copolymer, which led to a less ordered structure, altered pore shape, thicker silica walls, and higher SSA and V_t_. Prolonging the reaction time from 1 h to 3 h at 40 °C did not cause significant differences in the XRD patterns but led to higher SSA and V_t_ of the silica product. However, the synthesis carried out for 4 h at 100 °C resulted in smaller SSA and V_t_ for the final SBA-15. Interestingly, the attempt to synthesize SBA-15 under the same conditions (time and temperature) but without the use of ultrasounds ended in failure.

El-Fiqi and Bakry [23] presented a rapid one-pot synthesis of spherical mesoporous silica by simply dropping TEOS into a methanol/NH_4_OH solution of CTAB during sonication and stirring. TEOS:CTAB:NH_4_OH:CH_3_OH molar ratio was 0.125:0.07:4.1:18.5, respectively. After 2 h of the synthesis, the residues were removed, and the formed SiO_2_ particles were heated at 600 °C for 5 h under an air flow. The authors suggested the mechanism of the synthesis, which is illustrated in Figure 3B. In brief, applying ultrasounds induced free radicals of H^•^ and OH^•^ from H_2_O, which further initiated hydrolysis and condensation reactions of TEOS. SEM images showed spherical morphology, whereas TEM images revealed worm-like disordered mesopores in the as-prepared silica. Nitrogen adsorption isotherms indicated the presence of both micro- and mesopores in the silica structure; the calculated SSA was as high as 1544 m^2^/g, the V_t_ was 0.75 cm^3^/g, and the pore size ranged between 2 and 20 nm. Thermogravimetric analysis (TGA) indicated good thermal stability of the silica spheres. As evidenced in these works, the facile and fast sonochemical treatments could successfully replace time-consuming hydrothermal processes, which are typically used in the synthesis of mesoporous silicas. Apparently, free radicals generated in solvents and high-temperature regions induced by acoustic cavitation facilitate hydrolysis rate and condensation reactions between adjacent molecules of silica precursors (i.e., TEOS). Therefore, the ultrasonic irradiation helps optimize synthesis conditions, e.g., significantly reducing the synthesis time from days to hours. Moreover, ultrasonic treatments in an alcoholic solution could be utilized to remove surfactant templates from the resulting silica.

Fan et al. [24] obtained ordered mesoporous three-dimensional cubic FDU-12 with a spherical hollow structure using TEOS as a silica source, Pluronic F127 (EO_106_PO_70_EO_106_) as a structure directing agent, and TMB (1,3,5-trimethylbenzene) as a swelling agent. The reaction mixture with the formed micelles was sonicated at a power of 100 W in air for 6 h. Air bubbles generated by cavitation were covered with micelles, thus acting as templates for hollow structures. Then, the added TEOS interacted with the micelles, and upon the subsequent sonication (same conditions but for 8 h), its hydrolysis and condensation were accelerated. Finally, the template-containing product was calcined at 550 °C for 6 h to remove the polymeric template. Using the optimal molar ratios of reagents, i.e., TEOS/F127 of 252, TMB/F127 of 105 and KCl/F127 of 422 afforded FDU-12 silica with hollow structure, well-ordered cubic mesostructure (Fm3m), SSA and V_t_ up to 958 m^2^/g and 0.94 cm^3^/g, respectively, and a narrow pore size distribution (~15.5 nm). This study indicates that the generated bubbles can simultaneously act as the organizing agent for the self-assembly process of micelles and as an intrinsic template for hollow structures. On the other hand, the formation of organized polymeric or surfactant micelles is essential to mediate the interfacial tension and thus to stabilize the in-situ generated gas bubbles in the liquid phase.

The numerous reports available show the possibility of functionalization of silica surfaces with the use of ultrasounds. For example, under ultrasonication conditions, silica gel was successfully grafted by aminopropyltriethoxysilane (APTES), and the as-prepared product was examined for aldol condensation of furfural and acetone [25] or functionalized by different organic groups and tested for Suzuki coupling with aromatic halides [26]. Nevertheless, this review is focused on ultrasound-assisted synthesis rather than modification of pre-synthesized nanoporous materials.

Periodic mesoporous organosilicas (PMOs) are a class of silica-based hybrid materials usually synthesized via the hydrolysis and co-condensation of organosilica precursors (e.g., silsesquioxanes denoted as (RO)_3_Si-R-Si(OR’)_3_) and surfactants used as templates in either an acidic or basic environment [27]. PMOs have attracted much research attention because of the typical combination of a mesoporous silica-based framework and organic functionalities within pore walls. Organic bridges that have been successfully located in the pore walls of PMOs include simple ones such as ethylene (-C_2_H_4_-), ethyne (-C_2_H_2_-), methylene (-CH_2_-), and phenylene (-C_6_H_4_-), as well as more complex ones, such as aromatic, nitrogen- and sulfur-containing, ionic, and chiral groups, metal complexes, and many others [28]. Despite the huge variety of organic groups that could be incorporated, the use of the sonochemical method in the synthesis of PMO has hitherto been limited. The first successful sonochemical synthesis of PMOs was reported by Mohanty et al. [29] in 2010, in which a solution of P123 and a proper precursor, namely bis(triethoxysilyl)-methane (BTSM) for methane-bridged PMO (Me-PMO) and bis(triethoxysilyl)ethane (BTSE) for ethane-bridged PMO (Et-PMO), were sonicated for 30 min. After template extraction with acetone and HCl solution, the final Me-PMO and Et-PMO had SSAs of 1390 m^2^/g and 1201 m^2^/g, respectively. Kao et al. [30] used a similar ultrasound-assisted method to prepare benzene-bridged mesoporous organosilicas from 1,4-bis(triethoxysilyl)benzene (BTEB) precursor. The synthesis involved ultrasonic irradiation for 1–12 h and hydrothermal treatment for 0–12 h, followed by two-step calcination at 200 °C for 3 h and further at 300 °C for 3 h. The resultant materials showed well-ordered hexagonal mesostructures with SSAs in the range of 653–1097 m^2^/g. Two years later, Kao’s group [31] presented a modified procedure for the synthesis of benzene bridged PMOs using the reduced sonication time of 5 min. The modification relied on using a mixture of BTEB and TEOS, which afforded a highly porous product with an SSA of 1237 m^2^/g. Later, Rekha et al. [32] adopted the sonochemical method to obtain cyclophosphazene-bridged mesoporous organosilicas (CPMO) by co-condensation of APTES and phosphonitrilic chloride trimer (PNC), used as a source of additional organic groups. Changing the molar ratios of TEOS to the other components of the reaction mixture resulted in samples with SSAs varying from 305 to 974 m^2^/g after sonication for 1 h and the subsequent template removal with methanol and HCl. The as-obtained organosilicas were tested for dyes and Cr(VI) removal, achieving maximum adsorption capacities (at 25 °C) of 523 mg/g, 320 mg/g, and 101 mg/g for methyl orange, Congo red, and Cr(VI) ions, respectively. Apparently, ultrasonic irradiation not only accelerates the dissolution of precursors and assures a good homogenization of the synthesis solution but also facilitates the reactions between the uniformly distributed substrates: precursors and templates, which in turn influence the pore ordering and uniformity of the resulting mesoporous silicas and organosilicas. This may be the reason for the enhanced porosity of some sonochemically synthesized products. SSA values and potential applications of porous silicas and organosilicas obtained via sonochemical-assisted methods are summarized in Table 1 [20,21,22,23,24,25,26,27,28,29,30,31,32,33,34,35,36,37].

### 2.2. Metal Oxides

Ultrasound treatments have been widely used in the preparation of diverse metal oxides for various applications. Most of them exhibited low porosity, with SSAs below 100 m^2^/g. However, some papers report metal oxides synthesized via an ultrasound-assisted strategy and possessing relatively high porosity, such as TiO_2_, SnO_2_, CuO, MnO_2_, Fe_2_O_3,_ and Al_2_O_3_, and those will be discussed in this review. For instance, Zhang and Yu [39] reported the synthesis of hierarchical porous titania (HPT) spheres via a 3 h sonochemical treatment of titanium isopropoxide (TIP), pluronic P123, glacial acetic acid, and ethanol. The as-collected and dried powder at 100 °C in an oven showed meso- and macroporous structure and an SSA as high as 622 m^2^/g. The preparation of HPT under ultrasound consisted of three main stages, namely, the formation of spherical sol particles, their agglomeration, and then inter-agglomeration to produce mesoporous titania spheres. TEM images confirmed the evident connections between as-obtained mesoporous spheres, which the authors attributed to the effect of the shock wave generated by acoustic cavitation compacting the particles at a sufficiently high speed. Additionally, the ultrasound-induced high-temperature regions facilitated condensation reactions between the hydroxyl groups on adjacent particles and consequently the formation of agglomerates. Calcination of the as-prepared material decreased its surface area to 145 m^2^/g. Nevertheless, the calcined sample showed a high photocatalytic activity for the degradation of volatile organic compounds in air.

A synthesis under ultrasound waves was successfully employed to prepare highly porous tin oxide (SnO_2_) with SSA up to 433 m^2^/g [40]. The procedure involved the three-hour sonication of a solution of tin ethoxide (an inorganic precursor) and cetyltrimethylammonium bromide (an organic structure-directing agent) at pH 10. CTAB removal was carried out under optimized conditions, i.e., extraction for 24 h and calcination at 350 °C, leading to the sample with the highest SSA value among samples studied. The synthesized SnO_2_ samples showed different performances when used as electrodes in dye-sensitized solar cells, which mostly depended on the applied calcination temperature. The highest photocurrent values of 5.0 mA/cm^2^ were measured for the sample calcinated at 450 °C [40]. An analogous procedure was implemented for the synthesis of γ-Fe_2_O_3_, replacing the inorganic precursor with iron (III) ethoxide [41]. In this approach, CTAB was removed by 12-h extraction with ethanol, yielding nanoporous iron oxide with an SSA of 274 m^2^/g and good catalytic properties in the oxidation reaction of cyclohexane (i.e., a conversion of 36.5%). Yu et al. [42] prepared SnO_2_ particles inside mesoporous carbon via sonication of pre-synthesized mesoporous carbon (MC) and a SnCl_2_/ethanol solution without any other agents. An ultrasound treatment was performed at room temperature for 6 h, followed by washing, drying, and heating at 450 °C under an N_2_ atmosphere for 3 h. The obtained SnO_2_-MC composite had an SSA of 362 m^2^/g and exhibited about 186% higher capacity for lithium-ion storage compared to the initial mesoporous carbon framework, i.e., 200 mAh/g after 300 cycles.

Ávila-López et al. [43] prepared CuO with an SSA of 351 m^2^/g by using copper acetate hydrate (Cu(CH_3_COO)_2_·xH_2_O) as the metal oxide precursor and sodium citrate (Na_3_C_6_H_5_O_7_) as a surfactant. A sonochemical treatment was performed at 100 W for 15–60 min followed by washing and drying at 80 °C. The as-prepared CuO was examined as a CO_2_ adsorbent and a photocatalyst simultaneously, i.e., it can adsorb CO_2_ and photocatalytically decompose it to methanol with an efficiency as high as 7.4 mmol of CO_2_ per g of the adsorbent and 3.7 μmol/(g·h) in the CH_3_OH production process. Furthermore, such good CO_2_ adsorption and photocatalytic properties showed samples with low SSAs of 93 and 68 m^2^/g, prepared after 15 and 25 min of sonication, respectively, and without the addition of surfactant. The high CO_2_ uptake in the samples with relatively low porosity can be attributed to their favorable morphology of rectangular-shaped particles with edges that provide active sites for effective CO_2_ adsorption.

**Figure 4:**
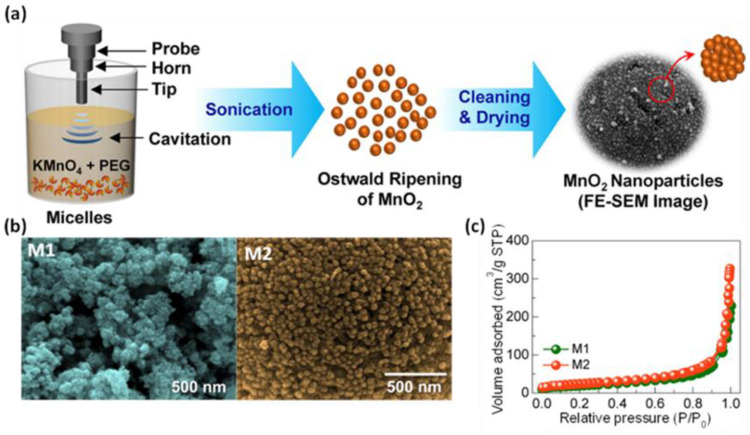
(**a**) Schematic illustration of synthesis of MnO_2_ nanoparticles via a sonochemical route, (**b**) SEM images, and (**c**) nitrogen isotherms of samples irradiated for 15 min (M1), and for 30 min (M2) [49]. Reproduced with permission from ref. [49]. Copyright 2018, Elsevier Ltd. and Techna Group S.r.l.

Majhi et al. [44] investigated the effect of different methods of boehmite sol treatments, i.e., using ultrasounds, conventional heating under reflux at 85 °C, and aging at room temperature, on the structural characteristics of the resulting boehmite samples and γ-aluminas obtained after calcination at 600 °C in a flow of air. For this purpose, they first prepared boehmite sols using aluminum chloride hexahydrate, ammonium hydroxide solution, and water as starting materials. The subsequent aging of boehmite sol under ultrasound followed by washing and drying at 80 °C gave a boehmite sample with an SSA of 256 m^2^/g, a V_t_ of 0.39 cm^3^/g, and an average pore diameter of 6.06 nm, while after calcination, the obtained γ-Al_2_O_3_ showed an SSA of 351 m^2^/g, a V_t_ of 0.29 cm^3^/g, and a pore size of about 3.23 nm. Applying conventional heating and aging at room temperature resulted in lower porosity for both calcinated (SSA of 325 m^2^/g and 201 m^2^/g, respectively) and non-calcinated (SSA of 219 m^2^/g and 191 m^2^/g, respectively) samples. Crystallite size calculations based on XRD analysis showed that γ-Al_2_O_3_ obtained by sonication had a smaller crystallite size compared to those obtained by the other methods. Apparently, the applied ultrasound treatment provided enough energy to break up agglomerates during aging. Sonochemically synthesized manganese oxides (MnO_2_) exhibited variable porosity, as evidenced by SSA values ranging from 168 to 301 m^2^/g [45,46,47,48,49]. MnO_2_ with the highest SSA (301 m^2^/g) was obtained by Zolfaghari et al. [45] from an aqueous solution of potassium bromate and manganese sulphate, which were irradiated with ultrasounds at 45 °C for nearly 9.5 h. Shortening the reaction time to 4 h resulted in a decreased in SSA of almost half (161 m^2^/g). Interestingly, the material with lower porosity showed a superior specific capacitance compared to the more porous sample, namely 344 F/g and 257 F/g, respectively. The synthesis of MnO_2_ in a significantly shorter time was proposed by Zuo et al. [46]. In brief, the aqueous solution of KMnO_4_ and Mn(CH_3_COO)_2_·4H_2_O was exposed to ultrasonic irradiation for only 20 min. The final product showed an SSA of 269 m^2^/g and promising properties for the oxygen reduction reaction (ORR). Sun et al. [48] proposed an interesting method for the synthesis of mesoporous MnO_2_ using only potassium permanganate and ethanol; this two-component mixture was sonicated at 25 °C for 1 h, resulting in a nanoporous MnO_2_ with an SSA of up to 192 m^2^/g and a narrow pore distribution with an average pore diameter of 10 nm. Apparently, during the synthesis process, the microbubbles formed due to the cavitation acted as a template to generate mesopores. The material was tested as supercapacitor electrodes, showing a specific capacitance of 229 F/g and retention of 97.3% after 2000 cycles. Sankar et al. [49] presented a synthesis of spherical α-MnO_2_ nanoparticles using potassium permanganate and polyethylene glycol as a structure-directing agent. The ultrasonic irradiation process was performed at 80 °C for 15 min (sample M1) or 30 min (sample M2). Extension of the reaction time was conducive to the formation of spherical particles. Moreover, sample M2 showed higher SSA (168 m^2^/g) and enhanced electrochemical capacity (136 F/g) compared to sample M1 (85 m^2^/g and 76 F/g, respectively). A schematic illustration of the synthesis procedure, SEM images, and nitrogen isotherms of M1 and M2 samples are presented in Figure 4. These works imply that the ultrasound treatment has a beneficial impact on the morphology and porosity of the resulting metal oxides, such as particle size and shape, pore size, and pore volume, as well as being very useful for the preparation of metal oxide-containing composites. SSA values and potential applications of porous metal oxides obtained via sonochemical-assisted methods are summarized in Table 2 [39,40,41,42,43,44,45,46,47,48,49,50,51,52,53,54,55,56,57,58,59,60,61,62,63,64,65,66,67,68,69,70,71,72,73].

### 2.3. Carbons

**Figure 5:**
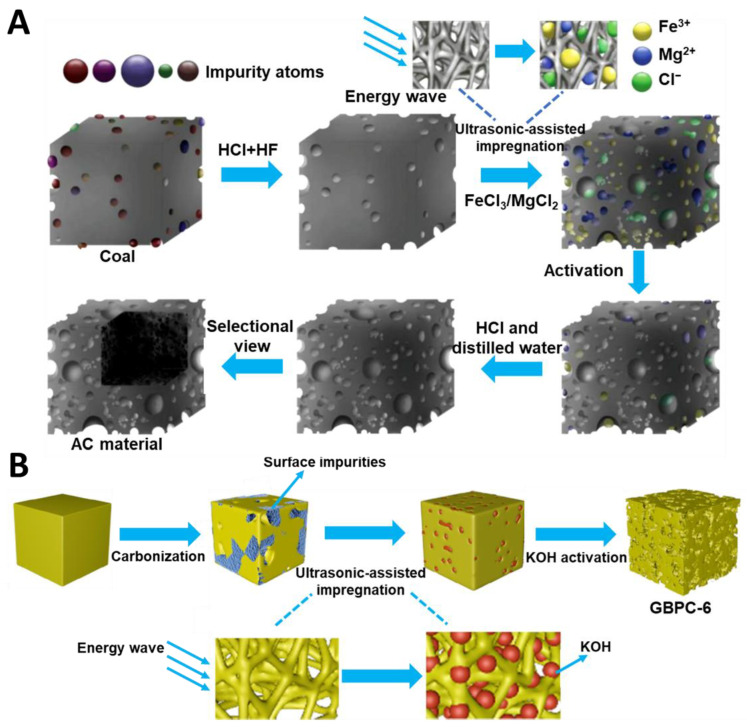
(**A**) Schematic illustration of activated carbon synthesis via ultrasound-assisted bimetallic activation strategy [75]. Reproduced with permission from ref. [75]. Copyright 2020, Elsevier B.V. (**B**) Schematic illustration of sonochemically synthesized garlic peel-based 3D hierarchical porous carbons [76]. Reproduced with permission from ref. [76]. Copyright 2019 Elsevier B.V.

Recently, green synthesis has been attracting a lot of attention, which has resulted in exploring the use of biomass and organic wastes as carbon precursors, mild activators, nontoxic chemicals, and non-conventional energy sources. The intention of green synthesis is to reduce environmental pollution, simplify synthesis procedures, and improve time- and cost-effectiveness. Altaf et al. [74] conducted an economic analysis of the synthesis of tea biochar-derived carbons, which relied on a sonochemically-assisted one-step pyrolysis. The estimated total cost per kilogram was about 77.9 USD. Moreover, the as-prepared sample (TUF_0.46_) exhibited mercury (Hg^0^) removal efficiency above 98% and high thermal stability at high temperatures. For comparison, the U.S. Environmental Protection Agency reports that the cost of removing mercury in coal-fired power plants with activated carbon is 500 USD per hour and the removal efficiency is about 90%. Such results showed that using both biomass and ultrasounds could effectively reduce the production cost of activated carbon (AC). Dong et al. [75] reported an AC prepared by using a mild ultrasound-assisted bimetallic activation strategy. Salts FeCl_3_ and MgCl_2_ were used as activators in a mass ratio to pre-cleaned coal (the carbon source) of 2:1. Such a mixture was mixed with distilled water and sonicated at 400 W for 0, 10, 20, or 30 min. Next, the dried samples were activated at 800 °C for 2 h under a CO_2_ atmosphere. The microscopic process of AC synthesis is presented in Figure 5A. The AC-20 sample (sonicated for 20 min) showed the highest SSA value of 2329 m^2^/g, micropore volume (V_mic_) of 0.92 cm^3^/g, and mesopore volume (V_mes_) of 0.40 cm^3^/g. Low content of impurities and high doping with oxygen (13.65 at.%) were crucial for the good electrochemical performance, i.e., 309 F/g at 0.5 A/g. Additionally, the authors noted that the use of ultrasounds resulted in a better distribution of activator molecules, which enhanced the pore formation process.

Sonochemically synthesized carbons could possess extremely high porosity, as evidenced by SSAs exceeding 3000 m^2^/g [76]. Dong et al. [75] used an ultrasonic-assisted impregnation to prepare physically modified garlic peel-based 3D (three dimensional) hierarchical porous carbons. Garlic peels were pre-carbonized at 600 °C for 2 h and then mixed with KOH, used as an activator, at a mass ratio of 4:1. The resulting mixture was sonicated at 65 °C for 0, 3, 6, or 9 min. The next step involved KOH activation at 800 °C for 2 h (Figure 5B). The as-synthesized carbons had SSA in the range of 3099–3887 m^2^/g and V_t_ in the range of 1.72–2.24 cm^3^/g, with the highest values relating to the sample obtained after 6 min of ultrasound treatment (GBPC-6). The non sonochemically-impregnated sample (GBPC-0) possessed lower SSA and V_t_ of 2548 m^2^/g and 1.58 cm^3^/g, respectively. Meanwhile, pore size distributions determined for all samples studied showed no changes, indicating that sonication is only a physical modification without causing pore structure collapse, which was also confirmed by other methods. SEM images revealed more abundant and regular pores in GBPC-6 compared to GBPC-0. Raman spectrum showed that ultrasonic waves made the carbon structure more ordered. According to XPS (X-ray photoelectron spectroscopy) analysis, the ultrasound-treated samples showed lower chemical adsorption of oxygen and a higher C/O ratio, which is beneficial for electrochemical performance, e.g., the specific capacitance increased from 304 F/g to 426 F/g at a current density of 1 A/g in a two-electrode test system. Apparently, the mechanical action of cavitation increases both the depth and velocity of liquid penetration into channels and pores of carbon, favoring a uniform distribution of an activator. In addition, a positive aspect of an ultrasound-assisted impregnation is associated with the efficient removal of impurities, which results in improved connectivity of the pore network.

**Figure 6:**
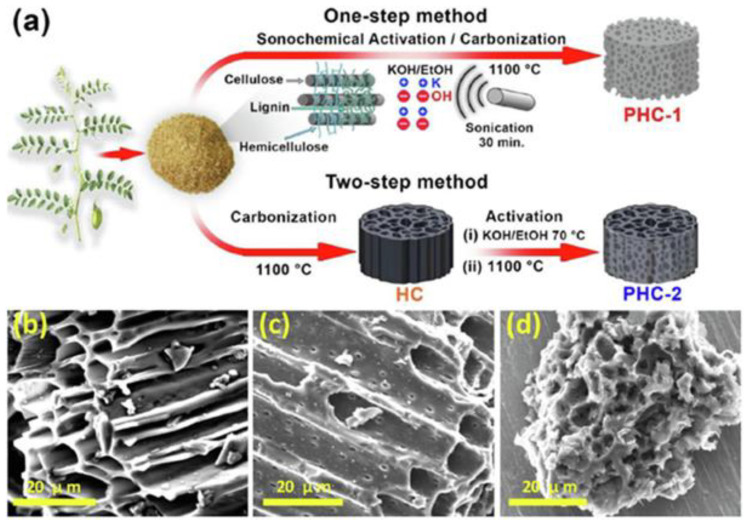
(**a**) Schematic illustration of both one-step sonochemical and two-step conventional synthesis of activated carbons. SEM images of (**b**) hard carbon (HC, nongraphitizable), (**c**) a conventional two-step activated sample (PHC-2), and (**d**) a sonochemical one-step activated sample (PHC-1) [77]. Reproduced with permission from ref. [77]. Copyright 2021, Elsevier Inc.

Recently, Ghani et al. [77] presented a comparison of a one-step sonochemical synthesis and a conventional two-step synthesis of a biomass-derived 3D porous hard carbon (PHC). Pre-treated raw chickpea husks (carbon source) and KOH (activator) were mixed in a weight ratio of 1:3. After the addition of deionized water, the mixture was sonicated at 1800 W (70 °C) for 30 min. Then, the dried powder was heated in a tubular furnace at 1100 °C for 2 h under an ambient atmosphere (Figure 6a). Such a procedure led to mesoporous carbon (PHC-1) with a SSA of 1599 m^2^/g and functional group contents of 8.1% for oxygen and 0.95% for nitrogen. A sample prepared via a conventional two-step activation method (without using ultrasounds, PHC-2) exhibited a lower SSA of 788 m^2^/g. SEM images showed differences in morphology depending on the synthesis method used; namely, a honeycomb-like structure was observed for the PHC-2 sample, while coral reef-such as morphology appeared when one step of sonochemical activation was applied (PHC-1). The morphology of PHC-1 suggests deeper perforation of metallic potassium into the cellulosic/hemicellulosic/ligninic chains of raw biomass, which was accomplished by using ultrasonic waves (Figure 6b–d). Carbon PHC-1 showed a high electrochemical performance, i.e., a specific capacity of 330 mAh/g at 20 mA/g, capacity retention of 89.5%, and structural stability over 500 sodiation/desodiation cycles at 1000 mA/g. This study shows that a facile ultrasonic irradiation can be effectively used to strengthen the chemical activation of carbon obtained from probably the most sustainable precursors, i.e., lignocellulosic biomass.

**Figure 7:**
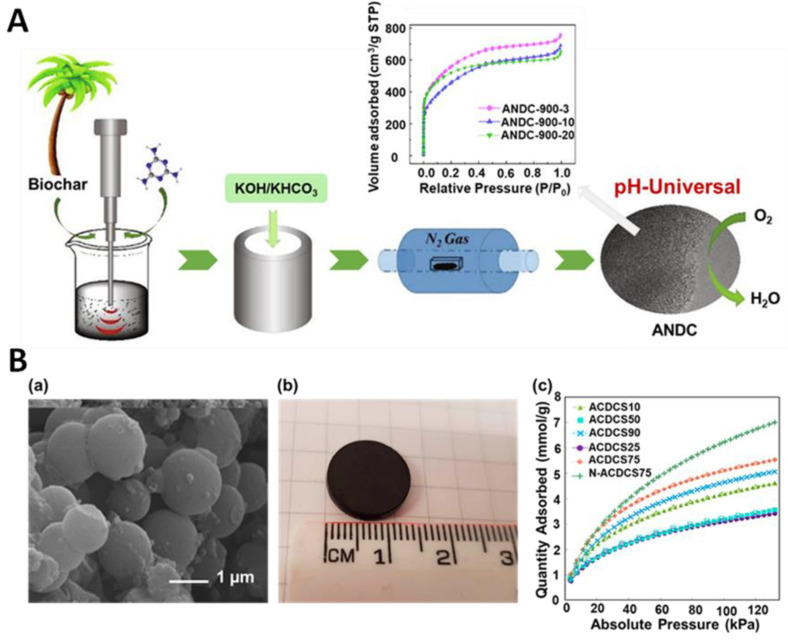
(**A**) Schematic illustration of a synthesis of biomass-derived activated carbons and nitrogen isotherms of the samples sonicated for 3, 10 and 20 min (ANDC-900-3, ANDC-900-10 and ANDC-900-20, respectively) [78]. Reproduced with permission from ref. [78]. Copyright 2020, Elsevier B.V. (**B**) (**a**) SEM images and (**b**) photograph of an activated carbon disc (ACD) prepared with 75% of carbon spheres. (**c**) CO_2_ adsorption at 0 °C on ACDs prepared with different content of carbon spheres [80]. Reproduced with permission from ref. [80]. Copyright 2019, American Chemical Society.

Another interesting approach to the synthesis of highly porous ACs is a combined strategy that involves different mechanical treatments. For instance, Wang et al. [78] presented an eco-friendly approach to synthesize coconut palm leaf-derived carbons using both ultrasound and mechanical treatments. The mixture of pre-prepared carbon precursor (washed, dried, and carbonized), melamine (a nitrogen source), and deionized water were sonicated at 200 W for 3, 10, or 20 min, leading to samples denoted ANDC-900-3, ANDC-900-10, and ANDC-900-20, respectively. After drying, these samples were ball milled with activators (KOH and KHCO_3_) under a vacuum at 300 rpm for 30 min, followed by pyrolysis at 300 °C for 2 h and heating at 900 °C for 2 h under nitrogen (Figure 7A). Depending on the sonication duration, the resulting samples exhibited different structural characteristics. For example, ANDC-900-10 had the highest density of graphitic-N and pyridinic-N groups per unit surface area, while ANDC-800-10 exhibited the highest SSA of 1949 m^2^/g. Moreover, it has been demonstrated that the oxygen reduction reaction performance of the carbon-based catalysts is closely related to the applied time of ultrasonication, activation temperature, nitrogen dopant, and activator used. Nevertheless, the authors implied that the as-obtained metal-free nitrogen-doped carbons are promising carbon-based catalysts for ORR at various pH levels. The carbonaceous catalysts aspire to be a good alternative to the commonly used noble metal catalysts.

In addition, there are reports on the synthesis of carbon spheres with the use of ultrasounds. Pol et al. [79] synthesized carbon spheres from resorcinol-formaldehyde (RF) resins via a rapid sonochemical route. The synthesis relied on ultrasound treatment of a solution of resorcinol, formaldehyde, ethanol, ammonia, and deionized water for 2–5 min. Then, the washed and dried spherical RF resins were carbonized at 600 and 900 °C, yielding microporous carbons with SSAs of 592 m^2^/g and 952 m^2^/g, respectively. The SEM images showed smooth surfaces of RF spheres with diameters of approximately 900 nm. Moreover, the authors studied the effect of temperature on the structural evolution of the carbon spheres using TEM, XRD, Raman spectroscopy, and Fourier transform infrared spectroscopy (FTIR). For example, the TEM image of single particles indicated that the surface of RF resins remained smooth after carbonization at 600 °C (CS-600), while surfaces became coarser for the samples heated at higher temperatures (CS-900 and CS-1100). Samples CS600 and CS900 were tested as supercapacitor electrodes and showed specific capacitances of 17.5 and 33.5 F/g, respectively. Gao et al. [80] reported the ultrasound-assisted synthesis of activated carbon discs (ACD) by assembling carbon spheres. At first, carbon spheres, ethanol, mesophase pitch, and KOH were mixed in different mass ratios, sonicated for 30 min in an ultrasonic bath, dried in an oven at 100 °C for 12 h, and ground using a mortar and pestle. Then, carbon discs of 14 mm diameter were formed, followed by carbonization in a tube furnace at 800 °C for 1 h under an argon atmosphere. The sample with the highest SSA of 1338 m^2^/g and V_t_ of 0.55 cm^3^/g was obtained from a mixture of carbon spheres:mesophase pitch mass ratio of 3:1 (ACDCS75). Such ACD had a bulk density of 0.62 g/cm^3^, a high compressive strength of 26.3 MPa, and CO_2_ and CH_4_ adsorption capacities of 6.64 and 5.82 mmol/g at 0 °C and 100 kPa, respectively (Figure 7B). Nitrogen-doped ACS (N-ACDCS75) prepared by post carbonization ammonia treatment showed high CO_2_ and CH_4_ uptakes of 8.23 and 6.24 mmol/g (at 0 °C and 100 kPa, respectively), indicating that the presence of N-containing functional groups enhances the strength of CO_2_ and CH_4_ interactions with the adsorbent compared to graphitic carbon sites. These studies imply that using ultrasounds can help carbonaceous materials acquire a preferred morphology and shape.

**Figure 8:**
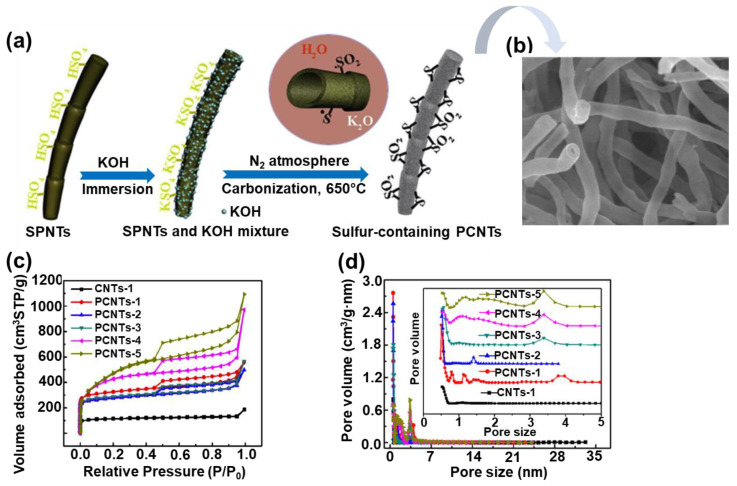
(**a**) Schematic illustration of synthesis of sulfur-containing porous carbon nanotubes (PCNTs). (**b**) SEM images of PCNT sample with sulfur content of about 3% (PCNTs-3), (**c**) nitrogen adsorption isotherms of PCNT samples, (**d**) pore size distributions determined for PCNT samples. Reproduced with permission from ref. [81]. Copyright 2017, Elsevier Ltd.

Furthermore, highly porous carbon nanotubes (PCNTs) were also successfully prepared under sonochemical conditions [81]. The procedure involved an ultrasound treatment of sulfonated polymer nanotubes (SPNTs) and KOH solution for 30 min and then heating at 650 °C for 1–5 h in nitrogen. A schematic illustration of the formation of sulfur containing PCNTs is presented in Figure 8. The sample obtained after 5 h of heating (PCNT-5) exhibited the highest SSA up to 1700 m^2^/g, a V_t_ of 1.69 cm^3^/g, and an average pore size of 3.98 nm. Nevertheless, the PCNT-3 sample showed the best electrochemical performance (331 F/g at 1 A/g), which can be attributed to the appropriate V_mic_ and V_t_ ratios (42%), which facilitated fast transport of electrons/ions and resulted in a high capacitance of the prepared CNT-based electrodes. Moreover, according to the XPS analysis, the PCNT-3 sample contained the greatest amount of sulfur, i.e., 2.97%, which is certainly not irrelevant for the afore-mentioned potential applications.

**Figure 9:**
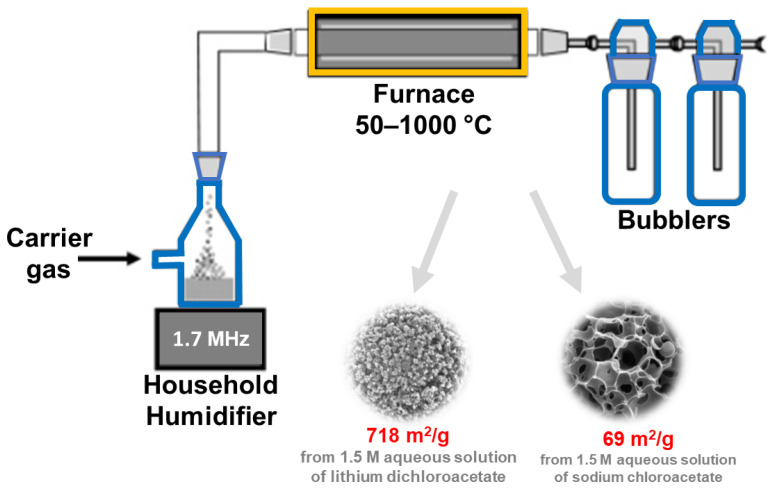
Schematic illustration of ultrasonic spray pyrolysis apparatus, and SEM images of exemplary carbons obtained using this technique. Reproduced with permission from ref. [84]. Copyright 2007, American Chemical Society.

It should be noted that, from an industrial viewpoint, the development of an efficient continuous production process for nanoporous materials is crucial. This can be achieved, e.g., by using the ultrasonic spray pyrolysis (USP) method [82]. In this approach, the role of ultrasounds is to nebulize the solution, which is then introduced into the furnace, where chemical reactions take place. There are several reports in the literature devoted to the synthesis of porous carbons using the USP method [82,83,84,85]. For example, Jung et al. [85] applied USP to prepare sucrose-derived activated carbon with SSA and V_t_ of 883 m^2^/g and 0.93 cm^3^/g, respectively. A spray precursor solution containing sucrose as a carbon source, Na_2_CO_3_ as a base catalyst for efficient decomposition of sucrose, and distilled water was atomized by an ultrasonic nebulizer at 1.7 MHz and then introduced into an electronic furnace (air flow, 800 °C). In the synthesis, the time counted from the bubble formation and passage of carrier gas to the end of the reaction tube (a length of 60 cm) was only 5 s; it is a so-called ultrafast flow. The collected product was washed with distilled water to remove any residual impurities/byproducts. The as-synthesized carbon was used to prepare the sulfur electrode for the sulfur-lithium battery, showing a high specific capacity of 1412 mAh/g_sulfur_. A schematic illustration of an ultrasonic spray pyrolysis apparatus is presented in Figure 9. SSA values and potential applications of porous carbons obtained via sonochemical-assisted methods are summarized in Table 3 [74,75,76,77,78,79,80,81,82,83,84,85,86,87,88,89,90,91,92,93,94,95,96,97,98].

### 2.4. Metal—Organic Frameworks and Covalent-Organic Frameworks

Nowadays, a great challenge for the world of science and business is to reduce greenhouse gas emissions, including carbon dioxide, which are responsible for global warming and extreme weather conditions. More and more new ideas are being implemented, including replacing conventional energy sources with more environmentally friendly ones (e.g., hydrogen fuel) or capturing and storing dangerous gases from pollutants emitted to the atmosphere (e.g., exhaust gases). MOFs are often considered solids with great potential to adsorb a variety of gases, mainly due to their huge specific surface areas and adjustable structure, among others. Jung et al. [100] compared the porosity and CO_2_ adsorption of MOF-177 (consisting of Zn_4_O clusters and 4,4′,4″-benzene-1,3,5-triyl-tribenzoate (BTB) linkers) prepared via three different synthesis methods, i.e., conventional solvothermal (C-MOF-177), sonochemical (S-MOF-177), and microwave (M-MOF-177). The adjusted optimal sonochemical conditions were 40 min of sonication at 60% power level (maximum 500 W at 20 kHz), which led to MOF with the highest SSA and V_t_ values of 4898 m^2^/g and 2.3 cm^3^/g, respectively, as well as a CO_2_ adsorption capacity of 29.89 mmol/g at 30 bar and 25 °C. Additionally, depending on the method applied, the prepared MOFs differed in the structural parameters, e.g., crystal sizes ranged from 0.5 to 1.5 mm for C-MOF-177, 5 to 20 µm for S-MOF-177, and 15 to 50 µm for M-MOF-177. Importantly, both ultrasound and microwave-assisted methods greatly reduced the synthesis time to 35–40 min compared with the 48 h required for the solvothermal method. It should also be noted that the yield of samples synthesized under ultrasound was up to 95.6%, while significantly lower yields of 66.7% and 71.1% were obtained for the solvothermal and microwave routes, respectively. This report clearly illustrates the substantial advantage of ultrasound-involved methods over other synthesis methods for MOFs.

It should be noted that the value of the specific surface area is not the only determinant of the adsorption capacity of MOFs towards adsorbate molecules such as carbon dioxide. CO_2_ adsorption is generally a weak physisorption process, which means that it is necessary to strengthen the adsorbate-adsorbent interaction, among others, by functionalizing the adsorbent surface, among others. Catenation, that is, the self-assembly of multiple separate frameworks within each other, may enhance the stability of the resulting supramolecular framework but may significantly diminish the porosity of the structure. Nevertheless, catenated MOFs are characterized by tunable pore sizes and enhanced adsorption selectivity, which are very favorable features in gas separation processes. Kim et al. [101] showed that control over the catenation process can be achieved simply by a proper adjustment of ultrasonic power (UP) levels during the sonochemical synthesis of CuTATB (TATB = 4,4′,4″-s-triazine-2,4,6-triyltribenzoate). The substrate mixture for CuTATB was subjected to different UP levels for 1 h, giving both catenated and non-catenated structures denoted as CuTATB-60 (60% of UP) and CuTATB-30 (30% of UP), respectively. The catenated sample showed better porosity (SSA of 3811 m^2^/g and V_t_ of 1.55 cm^3^/g) than the non-catenated one (SSA of 2665 m^2^/g and V_t_ of 1.07 cm^3^/g). According to TGA measurements, the catenated structure was more thermally stable. SEM images showed that CuTATB-30 consisted of small uniform particles with diameters in a range of 1.5–2 mm, whereas CuTATB-60 was composed of particles with diameters in a range of 4.5–6 mm. Catenation also had an effect on gas adsorption, as indicated by the CO_2_ uptake capacities of 3.54 and 4.29 mmol/g at 25 °C for CuTATB-30 and CuTATB-60, respectively. The selectivity of CO_2_/N_2_ was greater than 20:1 at 1 bar for both materials. Additionally, under high-pressure conditions (30 bar), the CO_2_ uptake on CuTATB-60 was up to 27.27 mmol/g.

**Figure 10:**
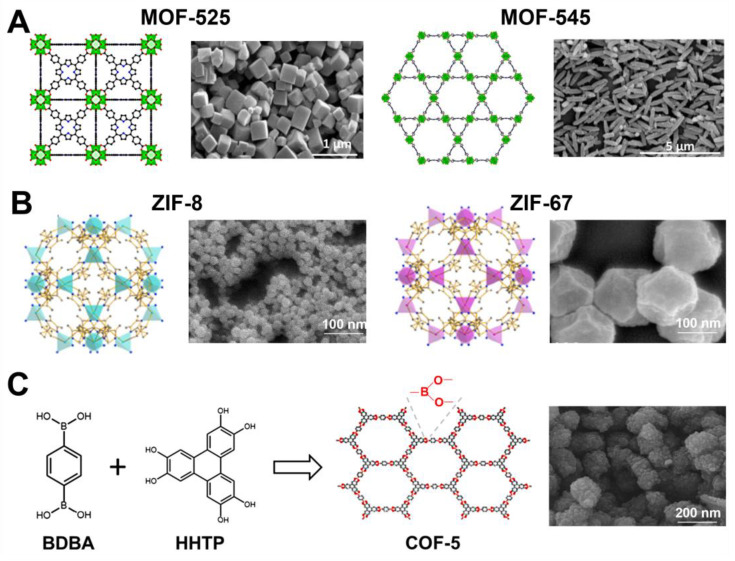
(**A**) Schematic illustration of structures and SEM images of MOF-525 and MOF-545. The green polyhedrons are Zr atoms, while the red, blue, and black spheres are oxygen, nitrogen, and carbon atoms [102]. Reproduced with permission from ref. [102]. Copyright 2021, Elsevier Inc. (**B**) Schematic representation of crystal structures of ZIF-8 and ZIF-67 (blue and purple tetrahedra represent tetrahedrally coordinated zinc and cobalt, respectively) [107]. Reproduced with permission from ref. [107]. Copyright 2020, Elsevier Inc. (**C**) Schematic illustration of synthesis of COF-5 and its SEM image [111]. Reproduced with permission from ref. [111]. Copyright 2018, Elsevier B.V.

Recently, Yu et al. [102] employed a sonochemical route for the synthesis of Zr-based porphyrinic MOF-525 and MOF-545, comprised of Zr_6_(OH)_4_O_4_(CO_2_)_12_ clusters and Zr_6_O_8_(CO_2_)_8_(H_2_O)_8_ clusters, respectively. The general procedure involved 30 min of ultrasound-assisted dissolution of substrate precursors (zirconyl chloride octahydrate and benzoic acid in dimethylformamide for MOF-525 and zirconyl chloride octahydrate and trifluoroacetic acid in dimethylformamide for MOF-545), then tetrakis(4-carboxyphenyl)-porphyrin (TCPP) was added to the solution, and a proper sonochemical treatment was continued. MOF-525 with the highest SSA (2557 m^2^/g) was obtained by sonication for 3 h under power conditions of 30% (i.e., a temperature of 102.2 °C). This material was composed of cubic/needle crystals with particle sizes in the range of 0.5–5 µm and a pore volume of 1.26 cm^3^/g. MOF-545 achieved SSA as high as 2248 m^2^/g and a pore volume of 1.77 cm^3^/g after 30 min of sonication under power conditions of 60% (i.e., temperature of 118.2 °C) followed by acid washing. It exhibited needle-shaped crystals with particle sizes in the range of 0.8–1.2 µm. Figure 10A shows the structures and SEM images of MOF-525 and MOF-545. Importantly, the porosity of both sonochemically synthesized MOFs was improved compared to the ones obtained via the conventional method, possessing SSAs of 1993 m^2^/g for C-MOF-525 and 1842 m^2^/g for C-MOF-545. The enlargement in SSA and V_t_ under ultrasounds can be attributed to the intensified creation of defect sites caused by a shortened time of nucleation and crystal growth. The enlarged amount of defects, larger SSA and V_t,_ resulted in better catalytic and adsorption performances. For instance, the adsorption of bisphenol-A (BPA) on S-MOF-545 and C-MOF-545 samples was 492.4 and 471.3 mg/g, respectively.

The synthesis of MOFs with desirable functional and structural properties faces some challenges, including the formation of undesirable topologies and the low solubility of the building blocks, among others. Linker exchange using pre-synthesized frameworks has been developed to overcome the above-mentioned drawbacks. The most often used approach is solvent-assisted linker exchange (SALE); however, long reaction times and green chemistry aspects became an incentive to look for new solutions. Razavi and Morsali presented an ultrasonic-assisted linker exchange (USALE) strategy [103]. The effectiveness of this method was tested by transforming the nonporous TMU-4 framework {with the formula [Zn(OBA)(BPDB)_0.5_]n·2DMF, in which H_2_OBA = 4,4′-oxybis(benzoic acid) and BPDB = 1,4-di(4-pyridyl)-2,3-diaza-1,3-butadiene} into the TMU-34 framework {with the formula ([Zn(OBA)(H_2_DPT)_0.5_]_n_·DMF, in which H_2_DPT = 3,6-di-(pyridyl)-1,4-dihydro-1,2,4,5-tetrazine)} by the replacement of BPDB with H_2_DPT. The first advantage of the applied approach was the significant reduction of the reaction time from 3120 min (for SALE) to 120 min (for USALE). Furthermore, gas adsorption studies (N_2_ at −196 °C and CO_2_ at 25 °C) showed that the USALE-synthesized TMU-34 showed larger porosity (SSA = 830 m^2^/g, V_t_ = 0.38 cm^3^/g) and higher CO_2_ adsorption capacity in comparison to the values for the TMU-34 samples obtained by both SALE (SSA = 720 m^2^/g, V_t_ = 0.34 cm^3^/g) and direct sonochemical (SSA = 540 m^2^/g, V_t_ = 0.28 cm^3^/g) methods. The authors claimed that the improved porosity of USALE-TMU-34 can be attributed to the intensified defect creation and particle size reduction induced by ultrasounds. However, USALE-TMU-34 showed better performances in other adsorption and catalytic experiments, e.g., removing a larger amount of Congo Red from an aqueous solution and reaching the maximum conversion in a Henry condensation reaction in a shorter time in comparison to other prepared TMU-34 samples [103].

ZIF-8 is a widely studied nanoporous material for many applications, including gas adsorption, catalysis, electrochemistry, and drug delivery. It belongs to zeolitic imidazolate frameworks (ZIF), a sub-class of MOFs composed of tetrahedral units where every metal ion (M = Zn or Co) is attached to four organic imidazolate linkers (Im) [104]. Their structure has been similar to that of zeolites, in which Zn or Co cations act as Si and imidazolate anions form bridges simulating the role of oxygen in zeolites, and even the angle of the M-Im-M bond is similar to that of the Si-O-Si bond. Therefore, they show the combined assets of both MOFs and zeolites, namely, high crystallinity and porosity along with great chemical and thermal stabilities. ZIF-8 consists of Zn and 2-methylimidazolate and displays a solidate (SOD) topology. Due to its stable structure and high porosity, it is a desirable material for a variety of applications. For instance, Ho et al. [105] synthesized highly porous ZIF-8 with SSA of 1832 m^2^/g for drug loading; Cho et al. [106] synthesized ZIF-8 with SSA of 1454 m^2^/g for the Knoevenagel condensation reaction; and Yao et al. [107] reported the same ZIF with SSA of 1414 m^2^/g for catalytic processes. The latter paper reports on the synthesis of bimetallic CoZn-ZIFs and ZIF-67 as well. The general procedure involved sonication of two separate methanol solutions of zinc nitrate hexahydrate and 2-methylimidazole, mixing them together, and sonication again for 16 min overall [107]. The co-doped ZIF was obtained analogously, except a Zn source was partly (in 25%, 50%, or 75%) or fully (for ZIF-67) substituted by the co-source. Figure 10B shows schematic structures and SEM images of ZIF-8 and ZIF-67. The addition of cobalt ions resulted in only a minor enlargement of SSA but doubled the degree of dye degradation in water by the obtained bimetallic MOFs. On the other side, ZIF-67 is chemically unstable; therefore, bimetallic materials gave the best results in the catalytic test compared to both ZIF-8 and ZIF-67, because the presence of Zn(II) in the framework ensured chemical stability and Co(II) enhanced the overall catalytic performance.

Reviews of sonochemically synthesized MOFs, describing the influence of the parameters of the synthesis on their physicochemical properties, among others, have been published elsewhere [108,109]. Sonochemical routes were also successfully adopted for the synthesis of covalent-organic frameworks; however, to our knowledge, there are only a few reports of sonochemical syntheses of COFs [110,111,112,113]. Yang et al. [110] were the first to obtain COF-1, which is constructed of planar six-membered B_3_O_3_ (boroxine) rings, and COF-5, which is constructed of 1,4-benzene diboronic acid (BDBA) and 2,3,6,7,10,11-hexahydroxytriphenylene (HHTP). The typical procedure relied on the dissolution of BDBA (for COF-1) or BDBA + HHTP (for COF-5) in a solution mixture of mesitylene and 1,4-dioxane, followed by sonication at 114 °C or 119 °C for 1 h. The optimized synthesis conditions resulted in an SSA of 732 m^2^/g and a V_t_ of 0.55 cm^3^/g for COF-1 and 2122 m^2^/g and 1.38 cm^3^/g for COF-5. Based on the same procedure, Duan et al. [111] synthesized COF-5, possessing a lower SSA of 965 m^2^/g, to prepare a mixed matrix (COF-5/Pebax-1657) membrane for CO_2_/N_2_ separation. The results indicate that a small addition of COF-5 (i.e., 0.4 wt%) to the membrane was enough to improve its CO_2_/N_2_ selectivity from 31.3 (for pure Pebax-1657) to 49.3. A schematic illustration of the synthesis of COF-5 and its SEM image is presented in Figure 10C.

Recently, Zhao et al. [113] synthesized a series of COFs (COF-1 to COF-7) via an ultrasound-assisted method by sonicating solutions of amine and aldehyde monomers in aqueous acetic acid for 60 min. The samples were subsequently extracted with methanol and dried under a high vacuum for 24 h. Detailed information on the reagents used and obtained samples can be found in ref. [113]. According to PXRD patterns, all the COFs showed good crystallinities. The as-prepared COFs exhibited high porosity comparable to the COFs obtained by other methods and often close to theoretical values. For instance, COF-1 had an SSA of 2059 m^2^/g, while the theoretical SSA for the COF is 2327 m^2^/g. Furthermore, the materials showed high photocatalytic activities for the hydrogen evolution from water, even better than that of their solvothermally synthesized counterparts. The highest H_2_ evolution rates of up to 16.6 mmol/(g·h) under visible light (λ > 420 nm) were obtained for COF-3 in the presence of ascorbic acid as a sacrificial electron donor and Pt as a cocatalyst.

Overall, the ultrasound-assisted synthesis of MOFs and COFs affects their crystallinity, crystal size, porosity, and even stability, which is particularly relevant considering their potential applications, e.g., in adsorption, separation, and catalysis. Moreover, sonochemical methods are often utilized for the preparation of MOF-containing composites, e.g., with graphene derivatives [114,115]. SSA values and potential applications of MOFs and COFs obtained via sonochemical-assisted methods are summarized in Table 4 [100,101,102,103,104,105,106,107,108,109,110,111,112,113,114,115,116,117,118,119,120,121,122,123,124,125,126,127,128,129,130,131,132,133,134,135].

## 3. Conclusions

Nowadays, simple and fast synthesis using waste as precursors, green energy, and a small amount or no solvents is highly desirable. As shown in this review, sonochemical synthesis has great potential to meet these expectations, but there are challenges that require further research. The sonochemical synthesis of nanoporous materials is a competitive method to other mechanochemical methods, but above all to a classical solvothermal synthesis. The greatest advantages of this approach include short synthesis times and environmental friendliness. In this review, we presented the basic principles of acoustic cavitation, the beginnings of the use of ultrasounds, the diversity of commercially available equipment, and a comparison of sonochemistry with other mechanochemical methods. This review presents primarily a summary of the achievements in the field of sonochemical synthesis of nanoporous materials such as silicas, organosilicas, metal oxides, carbons, metal-organic frameworks, and covalent-organic frameworks. Among them, there are highly porous ones, e.g., MCM-41 with SSA exceeding 1000 m^2^/g, biomass-derived activated carbon with an apparent SSA of 3887 m^2^/g, and MOF-177 with SSA of 4898 m^2^/g. These results are comparable with those obtained by other non-conventional or even conventional methods, suggesting that ultrasounds appear to be a powerful tool available for superior and more sustainable synthesis methods of advanced functional materials. For instance, it was demonstrated that the generated bubbles during ultrasonic irradiation can act as a supporting template for the generation of specific pore networks or even hollow structures. However, in practice, it is difficult to control the size of such a template based on the in situ-generated gas bubbles [136].

Sonochemical synthesis is based on the effect of acoustic cavitation, i.e., the generation, growth, and collapse of microbubbles, which deliver enough energy to initiate a chemical reaction, split or deagglomerate particles, and stir the solution. It is hard to predict a universal effect of ultrasound treatment and specifically the general mechanism of the reaction, which depends on the type of reactor used, the applied irradiation power and time, and most importantly, the substrates and solvents used, etc. Overall, the use of ultrasound significantly shortens the chemical reaction time due to the generated heat and the rapid formation of small particles and other reactive species, including radicals. Moreover, this method enables the carrying out of selective chemical reactions. The ultrasonic preparation of each material requires individual optimization of the synthesis conditions. So far, numerous types of porous materials have been successfully obtained under ultrasound-assisted conditions, and in this review, only the selected materials are discussed. For more details, including the synthesis of other materials, the readers are also referred to refs [137,138,139].

Despite the high potential of the sonochemical method for the synthesis of highly porous materials, there are not many studies devoted to this method. Only a few papers devoted to the syntheses of porous organosilicas, and COFs can be found in the literature. Thus, ultrasound-assisted synthesis requires future development to demonstrate its usability and versatility in the synthesis of diverse nanoporous materials. Furthermore, most of the already reported studies are small-scale. Scaling up sonochemical-assisted production is still a challenge. Apparently, only ultrasonic spray pyrolysis ensures a continuous process for potential large-scale production.

Overall, some successful attempts have been made in the exploration of sonochemistry for the synthesis of functional porous materials and their future applications. However, a particularly important aspect is the practical use of the sonochemical method for the synthesis of advanced nanoporous materials. The first issue refers to the large-scale fabrication of these materials. A reproducible, scalable, and cost-effective method for the synthesis of different groups of porous materials that could be as universal as possible is still missing. The second challenge in using sonochemical synthesis is the understanding of the occurring mechanisms and the in-depth structure−performance relationships. It is desirable to establish such a comprehensive framework based on already-published papers, especially for commercial purposes, but also for new researchers and students. Third, the key to further expanding the exceptional application areas of the specific groups of functional porous materials lies in associating their advantages with the practical requirements. For specific applications, knowledge of the influence of ultrasounds on the characteristics and properties of the resulting materials is necessary. Finally, it is often difficult to design and synthesize sustainable functional porous materials for specific applications.

In our opinion, good results on a laboratory scale will drive significant advances in the use of ultrasounds to synthesize a variety of porous materials on an industrial scale in the near future. The development of specialized equipment for large-scale synthesis is in demand. It is particularly important to ensure the continuity of the production process as well as its safe operation, e.g., by providing remote control over the course of chemical reactions and automatic safety and notification systems. Undoubtedly, ultrasound-assisted procedures are very promising research directions that can afford new functional nanoporous materials as well as improve the currently used synthesis procedures. Efforts should be made to improve synthesis procedures to obtain materials with comparable or even better properties in comparison to their counterparts synthesized by conventional methods. We believe that ultrasound-assisted technologies will continue to evolve and possibly even replace the currently widely used non-ecological methods. Researchers’ attention should be focused on improving synthesis procedures, scalability, and reducing synthesis costs.

## Figures and Tables

**Table 1:** Porous silicas and organosilicas obtained via sonochemical-assisted synthesis.

Sample	SSA [m^2^/g]	Application	Ultrasound Treatment	Ref.
**Silicas**				
MCM-41 *	1662 (1130)	Not reported	5 min/(200 W)/water	[20]
spherical mesoporous silica particles *	1544 (1014)	Not reported	20 min/(250 W)/methanol	[23]
MCM-41	1325	Not reported	30 min/60 °C/ethanol	[21]
MCM-41	1228	Catalytic cyclohexanone oxime conversion	30 min/(300 W)/water	[33]
FDU-12-T	958	Not reported	8 h/(100 W)/hydrochloric acid	[24]
MCM-41	931	Not reported	3.5 h/-/water	[34]
SBA-15	717	Not reported	1 h/80 °C/hydrochloric acid-water	[22]
colloidal nanosilica	369	Not reported	60 min/(30 W)/sodium hydroxide	[35]
nickel-silica catalyst	289	Hydrogenation of edible oil	10 min/-/-	[36]
manganese octahedral molecular sieve	267	Catalytic benzyl alcohol oxidation	3 h/55 °C/nitric acid-water	[37]
**Organosilicas**				
methane bridged PMO	1390	Not reported	30 min/25 °C/ammonia–water	[29]
benzene-bridged MCM-41	1237	Not reported	5 min/(200 W)/ammonia	[31]
ethane bridged PMO	1201	Not reported	30 min/25 °C/ammonia–water	[29]
benzene bridged PMO	1097	Not reported	1 h/85 °C(200 W)/hydrochloric acid-water	[30]
cyclophosphazene-bridged PMO	974	Adsorption of methyl orange, Congo Red and Cr(VI)	1 h/(300 W)/sodium hydroxide-water	[32]

* SSA values of the samples provided by the authors are overestimated because of the use of an incorrect range of relative pressures for the determination of SSA; for these MCM-41 materials the capillary condensation data partially overlap with the adsorption data used for calculation of SSA, which leads to its overestimation. The values of the geometrical surface area [m^2^/g] calculated using the relationship S = 4·10^3^ V_p_/w for cylindrical mesopores (V_p_—volume of ordered pores [cm^3^/g], w—diameter of ordered pores [nm]) are given in brackets [38].

**Table 2:** Porous metal oxides obtained via sonochemical-assisted synthesis.

Sample	SSA [m^2^/g]	Application	Ultrasound Treatment	Ref.
TiO_2_	622	Photocatalytic degradation of a volatile organic compound	3 h/-/ethanol-water	[39]
SnO_2_	433	Dye-sensitized solar cells	3 h/(100 W)/ammonia-water	[40]
SnO_2_	362	Lithium batteries	6 h/25 °C/sulfuric acid-ethanol	[42]
CuO	351	CO_2_ adsorption	37 min/(100 W)/water	[43]
MnO_2_	301	Supercapacitors	566 min/45 °C/water	[45]
TiO_2_–Al_2_O_3_	296	Not reported	4 h/40 °C (100 W)/ammonia	[50]
TiO_2_	292	Photocatalytic degradation of methyl orange	9 h/25 °C (40 W)/water	[51]
γ-Fe_2_O_3_	274	Catalytic conversion of cyclohexane	3 h/(100 W)/ethanol-ammonia-water	[41]
MnO_2_	269	Oxygen reduction reaction	20 min/(750 W)/water	[46]
MgAl_2_O_4_	267	Not reported	8 h/(750 W)/water	[52]
MnO_2_	245	Supercapacitors	30 min/40 °C (400 W)/ethanol	[47]
MnO_2_	192	Supercapacitors	1 h/25 °C (400 W)/water-ethanol	[48]
MnO_2_	168	Supercapacitors	30 min/80 °C/water	[49]
Mn-TiO_2_	162	Adsorption of aromatics	3.5 h/80 °C/ethanol-nitric acid	[53]
NiO	141	Supercapacitors	17 h/25 °C (60 W)/ionic liquid	[54]
mixed Ce, Zr oxides	132	Catalytic oxidation of formic acid	3 h/200 °C (750 W)/oleylamine	[55]
CeVO_4_	109	Electrochemical hydrogen storage	30 min/200 W/water	[56]
NiO	104	Not reported	3 h/25 °C/ethanol-water	[57]
NiO	103	Not reported	80 min/80 °C/water	[58]
ZnO	86	Photocatalytic degradation of organic pollutants	30 min/50 °C (60 W)/methanol-dimethyl formamide	[59]
CeO_2_	75	Electrochemical storage of hydrogen	15 min/(50 W)/hydrazine	[60]
Co_3_O_4_	72	Catalytic oxidation of hydrocarbons	3 h/70–80 °C/ethanol-water	[61]
Co_3_O_4_	70	Not reported	3 h/25 °C/ethanol-water	[57]
MoO_3_	55	Not reported	5 h/70 °C/water	[62]
MnO	53	Not reported	1 h/(45 W)/ethanol-water	[63]
Mn_2_O_3_	48	Not reported	3 h/(600 W)/water	[64]
ZnO	40	Not reported	1 h/(45 W)/ethanol-water	[63]
NiO	39	Catalytic oxidation of hydrocarbons	3 h/70–80 °C/ethanol-water	[61]
ZnO	37	Photocatalytic H_2_ generation	30 min/(150 W)/ethanol-water	[65]
ZnO	35	Not reported	3 h/-/water-dimethylformamide (DMF)	[66]
Cr_2_O_3_	35	Not reported	3 h/(600 W)/water	[64]
ZnO	34	Dye-sensitized solar cells	2 h/-/water–dimethylformamide	[67]
NiO	33	Not reported	1 h/(45 W)/ethanol-water	[63]
NiO	31	Lithium-storage	40 min/(100 W)/-	[68]
ZnO	25	Photocatalytic degradation of Rhodamine B and methyl orange	35 min/25 °C (60 W)/-	[69]
WO3	16	Triethylamine detection	(I) 1 h/(100 W)/hydrochloric acid- hydrogen peroxide (II) 2.5 h/(80 W)/hydrochloric acid- hydrogen peroxide	[70]
ZnO	16	Photo-remediation of heavy metal	2 h/25 °C/2-propanol-water	[71]
ZnO	15	Antibacterial applications	2 h/60 °C (200 W)/sodium hydroxide	[72]
ZnO	11	Antibacterial and anti-counterfeiting applications	2 h/50 °C (200 W)/sodium hydroxide-water	[73]

**Table 3:** Porous carbons obtained via sonochemical-assisted synthesis.

Sample/Description	SSA [m^2^/g]	Application	Ultrasound Treatment	Ref.
GBPC-6/garlic peel-derived activated carbon	3887	Supercapacitors	30 min/65 °C/potassium hydroxide	[76]
AC-20/coal-based activated carbon	2329	Supercapacitors	20 min/(400 W)/water	[75]
NCS-800/anionic polyacrylamide-derived carbon nanosheets	1962	Supercapacitors	10 min/-/water	[86]
S-ZC-800/ZIF-8-derived carbon	1955	Supercapacitors	2 min/25 °C (100 W)/methanol	[87]
ANDC-800-10/biochar-derived activated carbon	1949	Oxygen reduction reaction	10 min/(200 W)/water	[78]
HP-CNS/coffee wastes grounds-derived activated carbon	1946	Supercapacitors	-/-/dimethylformamide	[88]
AC-950/commercial activated carbon-derived activated carbon	1894	Supercapacitors	3 h/-/potassium hydroxide	[89]
SN-CMK-9/type of ordered mesoporous carbon	1725	Catalytic synthesis of higher alcohol from syngas	20 min/25 °C/ethanol	[90]
PCNTs-5/sulfonated polydivinylbenzene nanotubes- derived carbon nanotubes	1700	Supercapacitors	30 min/-/potassium hydroxide-water	[81]
PHC-1/raw chickpea husk-derived activated carbon	1599	Sodium-ion batteries	30 min/70 °C (1260 W)/potassium hydroxide-water	[77]
ACDCS75/activated carbon discs from carbon spheres and mesophase pitch	1338	CO_2_, CH_4_ adsorption	30 min/-/ethanol	[80]
Cu-AC/copper-activated carbon from spent activated carbon	1160	Adsorption of methylene orange and Congo Red	3 h/(1200 W)/water	[91]
1.0MCC800/cellulose-derived activated carbon	917	Adsorption of methylene blue	2 h/25 °C/water	[92]
CS900/resorcinol-formaldehyde resins-derived carbon spheres	952	Supercapacitors	4 min/25 °C/ammonia-ethanol-water	[79]
SN-CMK-8/type of ordered mesoporous carbon	919	Catalytic synthesis of higher alcohol from syngas	20 min/25 °C/ethanol	[90]
HPC/sucrose-derived activated carbon	807	Lithium−sulfur battery	-/-/water	[85]
Fe–C/sucrose-derived iron-impregnated carbon spheres	800	Catalytic Cr(VI) reduction	-/-/water	[93]
PC-I/lithium dichloroacetate-derived carbon	719	Electrocatalysts for fuel cells	-/-/water	[84]
PCNT-B/ carbon nanotubes	718	Supercapacitors	2 h/25 °C/1,2-dichloroethane	[94]
LiDCA/ organic salts-derived activated carbon	710	Not reported	-/-/water	[83]
CeFe6%(3/5)/MSWU700/ biomass straw-derived activated carbon	686	Hg^0^ adsorption	40 min/40 °C/water	[95]
FMCM-U/ foam-like carbon monolith	678	Supercapacitors	20 min/90 °C (100 W)/ethanol-water	[96]
Sn/CMK-3/ type of ordered mesoporous carbon	624	Lithium-ion batteries	2 h/25 °C/water	[97]
TUF_0.46_/ waste tea feedstock-derived activated carbon	196	Magnetic sorbent for Hg^0^ removal	2 h/80 °C/water	[74]
UMC/ sludge-derived activated carbon	131	Magnetic sorbent for Cr(VI) removal	30 min/(800 W)/-	[98]
OLCs/ paraffin oil-derived activated carbon	101	Supercapacitors	15 min/60 °C/dimethylformamide	[99]

**Table 4:** Metal-organic frameworks and covalent-organic frameworks prepared via a sonochemical-assisted synthesis.

Sample	SSA [m^2^/g]	Application	Ultrasound Treatment	Ref.
**Metal-organic frameworks**				
MOF-177	4898	CO_2_ adsorption	40 min/(300 W)/1-methyl-2-pyrrolidone	[100]
CuTATB-60	3811	CO_2_ adsorption	1 h/(300 W)/N,N-diethylformamide	[101]
S-MOF-5	3208 (Langmuir)	Not reported	30 min/100 °C/1-methyl-2-pyrrolidone	[118]
SIRMOF-60	2749	Not reported	1 h/(300 W)/N,N-diethylformamide	[101]
S-MOF-525	2557	Catalysis of dimethyl-4-nitrophenyl phosphate (DMNP) and adsorption of bisphenol A	3 h/(150 W)/dimethylformamide	[102]
S-MOF-545	2248	Catalysis of dimethyl-4-nitrophenyl phosphate (DMNP) and adsorption of bisphenol A	30 min/(300 W)/dimethylformamide	[102]
Ni-MOF	2021	Not reported	38 min/45 °C (200 W)/ethanol	[117]
ZIF-8	1832	Drug encapsulation	1 h/30 °C (90 W)methanol	[105]
S-CuBTC	1771	CO_2_ adsorption	1 h/145 °C (150 W)/choline chloride-1,3-dimethylurea	[118]
Mg-MOF-74(S)	1690	CO_2_ adsorption	1 h/(500 W)/dimethylformamide-ethanol- water	[119]
ZIF-67	1482	Catalytic Rhodamine B and peroxymonosulfate degradation	16 min/25 °C/methanol	[107]
ZIF-8	1454	Knoevenagel condensation reaction	-/(300 W)/dimethylformamide	[106]
Cu-BTCDMF	1430	Not reported	2 h/(300 W)/dimethylformamide	[120]
ZIF-8	1414	Catalytic Rhodamine B and peroxymonosulfate degradation	16 min/25 °C/methanol	[107]
ZIF-8	1285	Adsorption of Rhodamine B and methyl orange	10 min/(200 W)/ammonium hydroxide	[121]
ZIF-8	1249	Knoevenagel condensation reaction	1 h/(300 W)/dimethylformamide	[122]
A-ZIF-8	1221	CO_2_ adsorption	10 min/40 °C/2-methylimidazole	[123]
MOF-5	1203	Not reported	5 min/25 °C/dimethylformamide	[124]
Cu_3_(BTC)_2_(H_2_O)_3_	1156	Not reported	1 min/25 °C/dimethylformamide-ethanol-water	[125]
Cu_3_(BTC)_2_(H_2_O)_3_(60)	1100	H_2_ adsorption	1 h/(60 W)/dimethylformamide-ethanol-water-cupric acetate dihydrate	[126]
[Cu_3_(BTC)_2_]	891	H_2_ adsorption	20 min/-/ethanol-water	[127]
USALE-TMU34	830	Catalysis of nitroaldol and adsorption of Congo Red	160 min/25 °C/dimethylformamide	[103]
IL-ZIF-8	705	CO_2_ adsorption	6 min/40 °C/2-methylimidazole	[123]
Tb–BTC	678	Luminescence	20 min/25 °C/dimethylformamide	[128]
TMU-34-F	560	Detection of Al(III)	160 min/(300 W)/dimethylformamide	[129]
nano-[Cu(1,4-di(1H-imidazol-4-yl)benzene)]	417	CO_2_, CH_4_, and H_2_ adsorption	10 min/25 °C/ethanol-water-ammonia	[130]
HTMU-55	403	Henry reaction	60 min/(12 W)/dimethylformamide	[131]
TMU-55	400	Henry reaction	60 min/(12 W)/dimethylformamide	[131]
TMU-7	393	Congo Red adsorption	90 min/-/dimethylformamide	[132]
Cu-BTC	376	Adsorption of rifampicin	1 h/(350 W)/ethanol	[133]
[Zn(TDC)(4-BPMH)]_n_·n(H_2_O)	235	Adsorption of 2,4-dichlorophenol (24-DCP) and amoxicillin (AMX)	90 min/50 °C (30 W)/ethanol-water	[134]
Ni-MOF-2h	86	Supercapacitors	2 h/-/dimethylformamide	[135]
**Covalent-organic frameworks**				
COF-5	2122	Not reported	1 h/114 °C/mesitylene-1,4-dioxane	[110]
COF-1	2059	Photocatalytic H_2_ evolution	1 h/(550 W)/acetic acid	[113]
COF-2	1890	Photocatalytic H_2_ evolution	1 h/(550 W)/acetic acid	[113]
COF-3	1587	Photocatalytic H_2_ evolution	1 h/(550 W)/acetic acid	[113]
COF-5	1746	Photocatalytic H_2_ evolution	1 h/(550 W)/acetic acid	[113]
COF-4	1425	Photocatalytic H_2_ evolution	1 h/(550 W)/acetic acid	[113]
COF-6	1013	Photocatalytic H_2_ evolution	1 h/(550 W)/acetic acid	[113]
COF-7	940	Photocatalytic H_2_ evolution	1 h/(550 W)/acetic acid	[113]
COF-1	732	Not reported	1 h/119 °C/mesitylene-1,4-dioxane	[110]
TpPa-1/ Tp = 1,3,5-triformylphloroglucinol; Pa-1 = 1,4-phenylenediamine	127	Chromatography	1 h 5 min/15–25 °C/ethanol-tetrahydrofuran (THF)	[112]

## Data Availability

Not applicable.
